# The early retiree divests the health workforce: a quantitative analysis of early retirement among Canadian Registered Nurses and allied health professionals

**DOI:** 10.1186/s12960-019-0381-5

**Published:** 2019-07-05

**Authors:** Sarah Hewko, Trish Reay, Carole A. Estabrooks, Greta G. Cummings

**Affiliations:** 1 Level 3, Edmonton Clinic Health Academy, Faculty of Nursing, University of Alberta, 11405 87 Avenue, Edmonton, T6G 1C9 Canada; 2 Strategic Management and Organization, 4-21D Business Building, Alberta School of Business, University of Alberta, Edmonton, Alberta T6G 2R6 Canada; 3 Department of Applied Human Sciences, University of Prince Edward Island, 550 University Ave, Charlottetown, PE C1A 4N3 Canada

**Keywords:** Health human resources, Canada, Early retirement, Registered nurses, Allied health professionals, Logistic regression, Workforce planning

## Abstract

**Background:**

Early retirement (before age 65) is the norm among registered nurses (RNs) and allied health professionals (AHPs) employed in Canada’s public system. As a country whose population is rapidly aging, it is in Canada’s best interest to try and extend the work lives of RNs and AHPs.

**Objectives:**

(1) To test the predictive validity of our conceptual model of early retirement among publicly employed, Canadian RNs and AHPs and (2) to compare, across professions, model fit and factor significance

**Methods:**

We conducted multivariable logistic regression in two data sets, one consisting of 483 retired RNs and the other of 177 retired AHPs. The number of AHP respondents limited our ability to comprehensively test the model.

**Results:**

Eighty-five percent of RNs and 77% of AHPs had retired early. (1) Results indicate that 25% of variance in RN early retirement and 19% of variance in AHP early retirement was explained by included variables. (2) Organizational restructuring increased odds of early retirement by more than 100% among RNs and AHPs. Among RNs (but not AHPs), both financial possibility and caregiving responsibilities predicted early retirement at statistically significant levels, while a “desire to stop working” predicted retirement at or after 65 years of age.

**Conclusions:**

Clearly, there is much more to learn about RN and AHP pathways to early retirement. Further research, ideally research exploring the role of workplace characteristics, attitudes, and beliefs towards retirement and work-related factors, could deepen our understanding of the phenomenon of RN/AHP early retirement.

## RESUMÉ

**Contexte:**

Préretraite (avant 65 ans) est la norme parmi les infermières et professionnels paramédicaux (PPs) employés dans le système public canadien. Comme un pays avec un population qui vieillisse rapidement, c’est dans l’intérêt meilleur du Canada d’essayer de prolonger la vie professionnelle des infermières et PPs.

**Objectifs:**

1) Tester la validité predictive de notre modèle conceptual de préretraite parmi les infermières et PPs employés dans le système public canadien; 2) Comparer, selon les professions, l’ajustement du modèle et l’importance des facteurs.

**Méthodes:**

Effectué régression logistique multivariée dans deux ensembles de données, l’un composé de 483 infermières et l’autre de 177 PPs. Tous les participants avaient déjà pris leur retraite. Le nombre de répondants PP a limité notre capacité à tester le modèle de manière exhaustive.

**Résultats:**

Quatre-vingt-cinq pourcent des infermières et 77% des PP avaient pris préretraite. 1) Les résultats indiquent que 25% du variance en préretraite chez les infermières et 19% du variance en préretraite chez les PPs s’expliquent par les variables incluses. 2) La restructuration organisationnelle a augmenté les probabilités de préretraite de plus de 100% chez infermières et PPs. Parmi les infermières (mais pas les PPs), le possibilité financière et les responsabilités en matière de soins étaient tous deux significativement prédictifs de la préretraite alors qu’un « désir d’arrêter de travailler » était significativement prédictif de la retraite à l’âge de 65 ans ou après.

**Conclusions:**

Ç’est bien évident qu’il y a beaucoup plus à apprendre sur les chemin des infermières et PPs à la préretraite. Des plus amples recherches, explorant idéalement le rôle des caractéristiques du lieu de travail, des attitudes et des croyances vis-à-vis la retraite et des facteurs liés au travail, pourraient approfondir notre compréhension du phénomène.

**Most-clés:**

ressources humaines en santé; préretraite; infermières; professionnels paramédicaux; Canada; régression logistique; planification de la main-d’œuvre.

Canada is one of many countries confronted with the possibility of age-related labor shortages [1]. It has become increasingly necessary to develop a framework to incentivize retention of older workers in the labor market [2]. Few would disagree that delaying age of retirement, at a population level, could significantly reduce the magnitude of economic consequences resulting from population aging [1]. Delaying retirement is of particular importance in health care, which is affected in two ways by the aging of the population: first, per capita health expenditures and requirement for health services increase with age [3], and second, the health care workforce is also aging [4].

In this study, we examined early retirement among Canadian publicly employed registered nurses (RNs) and allied health professionals (AHPs). Both RNs and AHPs are essential to health care systems globally. RNs outnumber all other groups of health care professionals [5, 6]. We have defined AHPs as health professionals with an existing entry-level required education of a bachelor’s degree or higher (e.g., pharmacists, physiotherapists, dietitians). As a group, AHPs provide essential services at all points on the healthcare continuum; they are of particular value in the prevention, management, and treatment of chronic conditions [4].

We defined early retirement as “first” retirement (could be partial retirement) before the age of 65 years. This definition of early retirement was the most commonly reported in a systematic review of longitudinal studies that identified factors linked to nondisability early retirement [7]. Health professionals are more likely to retire earlier than those in the broader population; on average, publicly employed Canadian RNs and AHPs retire at 58.1 years and 59.4 years respectively (unpublished Canadian Longitudinal Study on Aging (CLSA) data), compared to 61.6 years among Canadian public sector employees (broadly) and 63.6 years among Canadian retirees (all sectors) [8].

## Background

We developed a conceptual model of early retirement among RNs and AHPs—see Hewko et al. [9] to view the complete model. The contents of the model were derived from the retirement literature (broad and profession-specific). We established its face validity through a series of purposive interviews with Canadian RNs and AHPs between 45 and 85 years of age. Elder’s [10] Life Course Perspective guided our conceptualization of retirement; notably, this perspective encourages interdisciplinarity and recognizes the value of micro-, meso-, and macro-level factors in the study of individual life courses [10]. This perspective is commonly applied in studies of retirement [11–15]. All identified factors were incorporated into one of eight categories of predictors of early retirement among RNs and AHPs. Categories included lifestyle and health, attitudes and beliefs, work-related, organizational factors, sociodemographics, broader context, and family and workplace characteristics [10]. In the analysis reported below, we were unable to empirically test all factors included in the conceptual model.

*Attitude and belief factors* linked to early retirement include the sense of being “tired of work” [16] and the desire for leisure time [16, 17]. Among the *organizational factors* connected with early employee retirement are as follows: (i) access to programs incentivizing retirement [16], and (ii) organizational restructuring [17, 18]. Many *sociodemographic* factors are identified predictors of early retirement including household income [19] and eligibility for retirement benefits [16] such as pension. Age [20–22], often considered a sociodemographic variable, is an obvious predictor of retirement; we have classified this under *broader context* in recognition that year of birth has implications unrelated to biological age (i.e., generational effects [23]). Last, *family* considerations impact upon timing of retirement—specifically, an agreement with one’s spouse [17] and caregiving responsibilities [18] positively predict early retirement.

The RN and AHP workforces are, in many ways, very similar. Both require a minimum baccalaureate level of education and both are female-dominated. Both groups are employed in diverse settings and both commonly provide their services to patients or clients in a shared workspace. Neither is frequently the “most responsible practitioner” in the care of their patients [24]. In general, the RN and AHP professions grant practitioners a comfortable, middle-class lifestyle. Yet, there are important differences between RNs and AHPs and their work-life, differences that may lead to systematic differences in their approaches to retirement. Most tangibly, the content and scheduling of RN and AHP work can differ significantly. RNs more often work elongated, rotating shifts outside of banking hours [25] and more frequently perform physically demanding tasks.

Professions included in the allied health workforce are diverse but do share characteristics relevant to the employee-employer relationship. For example, AHPs often provide ancillary services within multidisciplinary teams and may span work boundaries (e.g., across work units or across facilities) [26]. RNs are, in general, more likely to be associated with a single work unit or team than are AHPs. We suspect that there are sufficient differences in the work experiences of members of these professional groups to result in divergent approaches to retirement decision-making.

Gaining a better understanding of retirement decision-making among Canadian, publicly employed RNs and AHPs would facilitate the development and implementation of effective workforce policy (whether governmental or institution-specific) by health care leaders and policy-makers. Even a minor increase in average age of RN/AHP retirement could lead to significant benefits at the workforce level and, correspondingly, to improvements in availability and quality of health care for Canadians.

### Purpose and aims

Our study aims were to:

1. Test the predictive validity of our conceptual model of early retirement among publicly employed Canadian RNs and AHPs,
2. Compare model fit across professional groups and the magnitude of associations between individual predictors and early retirement, and
3. Identify and discuss implications for RN and AHP workforce policy.

## Methods

### Data

The first wave of the Canadian Longitudinal Study on Aging (CLSA) was the source of our data. Over 50 000 Canadians between 45 and 85 years of age participated in the CLSA study; data collection began in 2011. A complex sampling frame was employed to ensure rural and urban representation, sufficient response rate, national representation (respondents from all 10 provinces), and optimal age-sex distribution. Institutionalized individuals, persons living on First Nations reserves, and those unable to respond in English or French were not eligible to participate; this is not an exhaustive list of exclusion criteria. Data collection will continue in waves over a period of 20 years. Details on the CLSA, its protocols, purpose, and importance have been published and are available at https://clsa-elcv.ca/doc/511 [27].

### Sampling

Responses to occupation- and work-setting questions in the CLSA were recorded by CLSA staff as free text. Interviews were conducted either face-to-face or over the telephone (cohort-dependent). A single researcher (SH) reviewed these free-text responses (French and English) to identify publicly employed AHPs and RNs. See Table 1 for the list of included allied health professions; “other” includes an audiologist and a child life therapist. Many individuals reported profession as “nurse,” making it difficult to distinguish RNs from licensed practical nurses and nursing assistants and health care aides. Respondents’ were classified as RNs if (1) they specifically stated an occupation of registered nurse or (2) stated occupation was “nurse” and a level of education as baccalaureate degree or higher. Minimum entry-level education for RN licensing in Canada has changed over time. The timing of the transition from diploma-level preparation to baccalaureate-degree preparation has varied across provinces. As of 2011, more than 50% of Canadian RNs still had less than a baccalaureate level of education [28] (e.g., a diploma or associate degree).

**Table 1:** Distribution of representation from within the allied health professions

	*n*	%
Pharmacist	39	22
Social worker	55	31
Dietitian	16	9
Occupational therapist	19	11
Physiotherapist	35	20
Speech language therapist	11	6
Other	2	1
Total	177	100

The CLSA sample is, broadly, nationally representative. Only those participants who were already retired were included in the analytic sample: we identified a total of 793 publicly employed RNs and 366 publicly employed AHPs in the CLSA sample—308 RNs and 189 AHPs had not yet retired. Participant data were segregated into separate data sets to facilitate independent analysis by occupational group.

### Measures

#### Independent variables

We used data from multiple CLSA survey questions without manipulation: age, household income, and factors contributing to retirement (binary—yes/no). To identify factors contributing to retirement, CLSA staff posed the question “There are many reasons why people retire. Which of the following reasons contributed to your decision to retire?” Respondents could select any number of reasons as contributors. In this analysis, we used responses for the following options (as presented to respondents with name of variable in brackets): Retirement was financially possible (financial possibility); completed the required years of service to qualify for pension (pension eligibility); wanted to stop working (“tired of work”); wished to pursue hobbies or other activities of personal interest (pursuit of hobbies); employer offered special incentives to retirement (retirement (dis)incentives); organizational restructuring or job eliminated (organizational restructuring); providing care to a family member or friend (caregiving responsibilities). The CLSA questionnaires are available at https://www.clsa-elcv.ca/researchers/data-collection.

#### Dependent variable

As noted previously, we defined early retirement as retirement before age 65. Respondents were asked “How old were you when you first retired/partly retired?” We coded all responses of > 65 years as 0 and responses of < 65 as 1.

### Analysis

All analyses were conducted in Stata SE 13.1®. We explored distribution and variance of each variable; no outliers were identified. The amount of missing data was minimal. To evaluate correlations between variables, we ran Pearson correlations with a correction for multiple comparisons (Bonferroni) (see Table 2). Correlation coefficients of 0.7 or greater indicate a strong relationship between variables [29]; when two variables are strongly correlated, it may be of little value (and potentially detrimental) to include both in multivariate models. The largest correlation coefficient between our variables was between pursuit of hobbies and financial possibility (in the RN sample) (*r* = 0.47, *p* < .05).

**Table 2:**
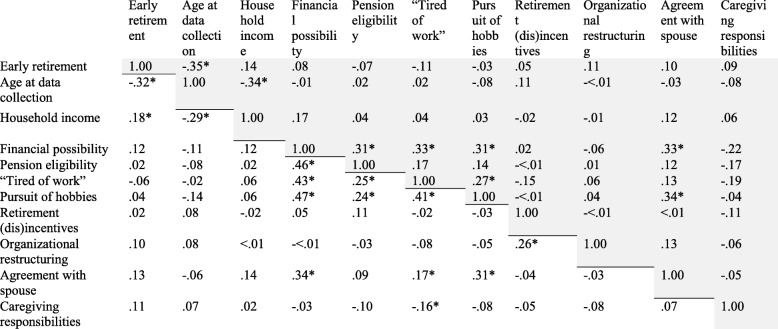
Correlation table (*n* = 483 RNs and *n* = 177 AHPs)

Correlations in RN sample are unshaded

Correlations in AHP sample are shaded

*p < .05

Variance inflation factor (VIF) is a commonly used measure of collinearity in regression models. A VIF > 10 is indicative of a problematic multicollinear relationship [30]. The highest VIF value in both the RN and AHP models was 1.78. Thus, we concluded that multicollinearity was not an issue in these models.

Sample size to adequately power a logistic regression is based on the number of “cases”; in this analysis, early retirees were considered “cases.” Peacock and Peacock [31] recommend a minimum ten cases per included variable. More recently, Austin and Seyerberg [32] reported that when cases per included variable are below 20, modern validation methods (e.g., bootstrapping) are recommended. Bootstrapping involves repeatedly drawing random samples from within the analytic sample [30]. We elected to be prudent and conducted bootstrapping when testing the model in both samples (RN and AHP).

**Fig. 1:**
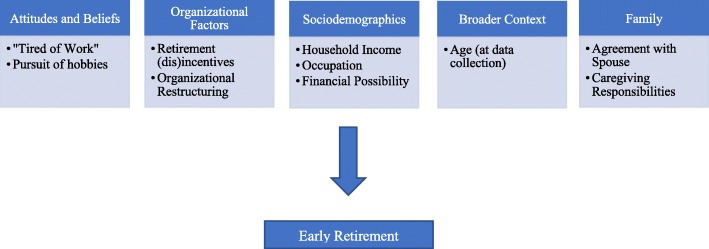
Analytic model of early retirement among registered nurses and allied health professionals

The power-limiting factor for our analysis was the number of early AHP retirees (*n* = 137). We selected 10 predictor variables from the original conceptual model [9] for inclusion as follows: (1) the two variables found to be significantly correlated with early retirement (household income and age at data collection) and (2) the eight variables directly connected to the retirement decision (i.e., the CLSA interview question began “Which of the following reasons contributed to your decision to retire?”). Including only ten predictor variables ensured that both models were adequately powered. We conducted an identical non-stepwise, unconditional, multivariable logistic regression in both occupation-specific data sets to test model fit (see Fig. 1).

## Results

Average age at data collection was 68.4 for RNs and 68.9 for AHPs (see Table 3). Eight-five percent of RNs and 77% of AHPs had retired before age 65. Several professions had significantly more respondents than the others—social work, pharmacy, and physiotherapy (see Table 1). Only 3% of RN respondents were male compared to 21% of AHPs. Among RNs, the most frequently reported factors contributing to retirement were financial possibility and “tired of work.” Both factors were also reported frequently among AHPs in addition to pension eligibility and pursuit of hobbies.

**Table 3:** Description of RN and AHP samples (*n* = 483 RNs and *n* = 177 AHPs)

	RNs (*n* = 483)	AHPs (*n* = 177)
Education level		
Less than bachelor’s degree	149 (31%)	26 (15%)
Bachelor’s degree	255 (53%)	92 (52%)
Post-bachelor’s education	79 (16%)	59 (33%)
Marital status		
Single	272 (56%)	68 (38%)
Married or living with partner	211 (44%)	109 (62%)
Gender		
Male	14 (3%)	38 (21%)
Age at data collection		
Mean (SD)	68.4 (7.7)	68.9 (8.4)
Province of residence		
Alberta	66 (14%)	17 (10%)
British Columbia	108 (22%)	48 (27%)
Manitoba	41 (8%)	15 (8%)
New Brunswick	7 (1%)	2 (2%)
Newfoundland and Labrador	34 (7%)	9 (5%)
Nova Scotia	30 (6%)	14 (8%)
Ontario	95 (20%)	40 (23%)
Prince Edward Island	10 (2%)	6 (3%)
Québec	72 (15%)	19 (11%)
Saskatchewan	20 (4%)	6 (3%)
Household income		
Less than $20 000	8 (2%)	1 (1%)
$20 000 to < $50 000	128 (29%)	33 (20%)
$50 000 to < $100 000	220 (50%)	84 (51%)
$100 000 to < $150 000	57 (13%)	27 (16%)
> $150 000	25 (6%)	19 (12%)
Early retirement	411 (85%)	137 (77%)
Factors contributing to retirement		
Financial possibility	218 (45%)	88 (50%)
Pension eligibility	147 (30%)	69 (39%)
“Tired of work”	212 (44%)	89 (50%)
Pursuit of hobbies	129 (27%)	70 (40%)
Retirement (dis)incentives	25 (5%)	13 (7%)
Organizational restructuring	57 (12%)	15 (9%)
Agreement with spouse	110 (23%)	40 (23%)
Caregiving responsibilities	73 (15%)	23 (13%)

The model explained 25% of variance in RN retirement timing and 19% for AHPs (*p* < .001). Organizational restructuring, as a contributor to retirement, significantly increased odds of early retirement among RNs and AHPs (Table 4).

**Table 4:** Logistic regression results (RN and AHP)

	RN model					AHP model				
	Odds ratio	Bootstrap standard error	*z*	95% CI lower	95% CI upper	Odds ratio	Bootstrap standard error	*z*	95% CI lower	95% CI upper
Constant	35 176.77	57 963.31	6.35*	1 392.08	888 884.70	10 156.86	22 202.72	4.22*	139.97	737 006.90
Age (at time of data collection)	*.86*	.02	− 6.44*	.82	.90	*.89*	.02	− 4.64*	.85	.94
Household income	*1.61*	.35	2.20*	1.05	2.47	1.00	.23	.01	.65	1.56
Factors contributing to retirement decision										
Financial possibility	*2.49*	.84	2.71*	1.29	4.82	2.35	1.24	1.61	.83	6.63
Pension eligibility	.68	.35	− .75	.25	1.87	.72	.32	− .073	.30	1.73
“Tired of work”	*.49*	.17	− 2.04*	.25	.97	.54	.38	− .88	.14	2.12
Pursuit of hobbies	1.05	.54	.10	.38	2.85	.64	.39	− .73	.19	2.13
Retirement (dis)incentives	1.40	.88	.54	.41	4.82	1.79	1.15	.91	.51	6.29
Organizational restructuring	*3.94*	2.68	2.02*	1.04	14.96	*5.59*	3.85	2.50*	1.45	21.58
Agreement with spouse	2.15	1.18	1.40	.74	6.28	1.85	1.30	.87	.46	7.34
Caregiving responsibilities	*7.60*	6.05	2.55*	1.59	36.17	2.68	2.75	.96	.36	20.03

RN model Log likelihood: − 137.91, Wald chi-square (10) = (*p* < .001), 43 replications, pseudo-*R*^2^ = .25

AHP model Log likelihood: − 71.71, Wald chi-square (10) = (*p* < .001), 26 replications, pseudo-*R*^2^ = .19

*p < .05

The italicized entries are the Odds Ratios associated with significant z scores

In the RN model, each incremental increase (one increment = $50 000) in household income was associated with a 1.61 times greater odds of retiring early. Among the factors reported by RNs as contributing to their retirement decision, financial possibility and caregiving responsibilities both significantly increased odds of early retirement. Alternately, those who indicated that being “tired of work” contributed to their retirement decision had significantly lower odds of having retired early.

## Discussion

Early retirement (before age 65) is the norm among publicly employed Canadian RNs and AHPs. We were able to test a pared-down version of a literature-derived conceptual model of early retirement: overall our tested model explained between 19 and 25% of variance in RN and AHP early retirement. As a country with existing shortages in health professionals (e.g., audiology and speech language pathology [33], pharmacy [34], occupational therapy and physiotherapy [33]) and an aging population, it is in Canada’s best interest to try and extend the work lives of RNs and AHPs.

It is unsurprising that much of the variation in early retirement remains unexplained by our model; we were unable to test associations of many meso-level variables (i.e., those identified in the categories of work-related, organizational factors and workplace characteristics in the complete conceptual model [9]) as these factors were not measured in the CLSA. The Life Course Perspective, used to guide our conceptualization of retirement, emphasizes the importance of micro-, meso-, and macro-level factors as influences of life course decisions.

Unfortunately, nearly all factors significantly associated with early retirement in our analyses are not easily mitigated or altered by health administrators or policy-makers. Although only 12% of RNs and 9% of AHPs reported that organizational restructuring contributed to their retirement decision, it was a significant predictor of early retirement among both RNs and AHPs. Previous research has demonstrated that the older workforce is at disproportionate risk throughout the process of organizational restructuring. There is a tendency to introduce and/or expand early retirement programs during restructuring in order to reduce the size of the workforce as the optics are better than those associated with sizeable layoffs [35]. Additionally, as restructuring is frequently triggered by financial constraints, reducing the number of older, more “expensive” [36] workers is seen as a way of reducing organizational expenses. In a unionized system, such that exists across Canada, administrators may seek to vacate positions by offering older workers incentivized early retirement packages.

Across Canada and the world, organizational restructuring is a common response to resource scarcity (actual or expected); as health care costs make up a significant part of many government’s budgets, the need for cost-cutting in health systems is unlikely to diminish. For nurses in particular, organizational restructuring commonly results in an increase in workload [37]. Early retirements triggered by organizational restructuring may exacerbate resource scarcity. Burke, Ng, and Wolpin [38] suggest that collaboration during implementation of change between hospital management and unions (such as nursing unions) may mitigate negative impacts on the employees in the organization.

Desire to stop working or being “tired of work,” which in this analysis was associated with significantly lower odds of early retirement among RNs, is a “catch-all” response worthy of further study. Respondents were specifically asked if wanting to stop working contributed to their decision to retire. They were given an opportunity to offer “other” reasons contributing to retirement but only two respondents in our analytic sample elected to do so. Without a better understanding of what it is specifically that made them want to stop working, it is difficult to develop strategies to alter this desire. It is possible that many of the meso-level factors not measured in the CLSA (but included in our conceptual model) such as frequent/pervasive change in the workplace and opportunities for flexible hours of work (or lack of) may have contributed to respondents’ desire to stop working.

In our analysis, caregiving responsibilities were associated with significantly higher odds of early retirement among RNs. The health professions are female-dominated, and according to US data, 60% of unpaid care providers are female [39]. In addition, female care providers are more likely than male care providers to provide caregiving as a reason for retirement [1]. Glenn [40] argues that for many women—whose roles often include mother, wife, and daughter—the duty to care is a role obligation.

Humble, Keefe, and Auton [41] noted that caregivers reported a willingness to remain in the paid workforce should circumstances be altered to facilitate their doing so. Strategies to subsidize caregiving support, expand leave policies to accommodate employees’ needs to provide care [42], and/or facilitate work flexibility [40] may be effective deterrents to early retirement. Glenn [40] also argues that, due to existing inequalities in the labor market that perpetuate gendered caring, affirmative-action policies and application of anti-discrimination laws could serve to equalize the cost of engagement in caring work for women.

It is clear, both from the number of literature-derived variables appearing in our model that were not measured in the CLSA and from the fact that our tested model left the majority of variance in the outcome of early retirement unexplained, that our understanding of retirement decision-making among publicly employed RNs and AHPs is far from complete. Future studies with a focus on measurement of workplace characteristics, attitudes, and beliefs and work-related factors would facilitate testing the influence of the remaining factors in our conceptual model on early retirement.

The diversity of AHPs in our sample likely contributed to the limited number of statistically significant relationships identified in the AHP model of early retirement. Although adequately powered to detect predictors of early retirement among AHPs in general, there were insufficient numbers of individual groups of professionals to test for differences across allied health professions. It is possible that a factor that contributed to higher odds of early retirement in one sub-group (e.g., pharmacists) contributed to lower odds of early retirement in another (e.g., social work) and thus sub-group analyses should be undertaken with a sufficient sample.

## Limitations

The limited AHP sample size bounded our capacity to test more factors associated with early retirement among RNs and AHPs. A more comprehensive survey, completed by a larger number of AHPs, in particular, would be required to test the complete conceptual model [9]. Additionally, our inability to confidently isolate all RNs in the sample limited the RN sample size. While the use of nationally representative samples in analysis can maximize the generalizability of results, it should be noted that the CLSA sampling frame was not designed to specifically ensure representativeness according to profession or setting of employment. For this reason, the AHP and RN samples may not be representative of the broader publicly employed AHP and RN population. There is, however, no indication that our sample is not representative—gender representation in the sample is in-line with proportions reported by the Canadian Institute for Health Information. In 2006, 94.5% of nurses were female [43] and, because the proportion of males in the profession is gradually increasing over time [44], it is likely that the proportion of those who are female that are over the age of 45 is greater (i.e., closer to our observed proportion of 97%). Pharmacists, however, a group with significant representation in our sample, were 59.8% female as of 2012 [45]; thus, it is expected that the AHP sample would have a larger proportion of male respondents. A future survey, designed explicitly to explore retirement decision-making in the RN and AHP workforce, should be administered using methods that maximize the representativeness of the sample with consideration for profession, setting of employment, province/region of residence, age, and gender.

The reference point (time-wise) for many of the variables was a limitation. For many questions, particularly those related to sociodemographics, participant responses represented their status at the time of data collection. It is possible that many respondents’ marital status, province of residence, etc. had changed since the time of retirement. Interestingly, many variables predictive of early retirement among RNs and AHPs, including financial possibility, “tired of work,” organizational restructuring, and caregiving responsibilities, were measured using questions that called on participants to think back to what had contributed to their retirement. Although there was potential for recall bias [31] when responding to these questions, these variables were more temporally connected to our outcome variable. Additionally, as we have analyzed only the first wave of data (cross-sectional) collected in the CLSA, we can only confirm associations (not causality) between independent variables and early retirement. In future, we hope to re-test the model using multiple waves of CLSA data.

We relied on free-text variables to assign participants to both an occupational category and a work setting; our goal was to ensure that only RNs and AHPs employed in the Canadian public health sector were included in the analytic sample. It is possible that participants were misallocated (either in or out of the analytic sample). An error may have occurred at either or both the data entry stage or during the selection process (conducted by SH). Due to space constraints, we were unable to discuss varied theories and conceptualizations of retirement and how the process may differ when retirement is early vs. delayed. We recommend Feldman [46], Shultz and Wang [47], and Fisher et al. [48] to those seeking in-depth theorization of retirement and timing of retirement.

The analytic method we have selected is not designed to test mediation effects. We have considered the magnitude and significance of correlations and tested for multicollinearity in our model. The literature review conducted to inform development of our conceptual model [9] did not identify specific factors as mediators in the relationships between predictors of retirement and early retirement. Theoretical or conceptual mediation effects would be better detected via other analytic methods, such as structural equation modeling.

## Conclusions

The majority of publicly employed, Canadian RNs and AHPs retire before age 65. Organizational restructuring, although cited by fewer than 15% of RNs and AHPs as a factor contributing to their retirement, increased odds of early retirement in this population. Health administrators may want to re-consider use of early retirement as a tool to achieve cost reduction during organizational restructuring. Although this strategy can help to improve the optics of system-wide change, it is a blunt tool that can exacerbate existing shortages in the health professions. Among RNs, both financial possibility and caregiving responsibilities predicted early retirement. The maximum explained variance in early retirement was 25% (among RNs) indicating that there is much to be learned about both RN and AHP pathways to early retirement. Further research, ideally exploring the role of workplace characteristics, attitudes, and beliefs and work-related factors (e.g., organizational tenure) in retirement decision-making among Canadian, publicly employed RNs and AHPs, would deepen our understanding of this phenomenon.

## Data Availability

The data that support the findings of this study are available from the Canadian Longitudinal Study on Aging (CLSA). Restrictions apply to the availability of these data—interested researchers can submit a data access application to the CLSA Data and Sample Access Committee (see https://www.clsa-elcv.ca/data-access/data-access-application-process).

## Abbreviations

AHP: Allied health professional
CLSA: Canadian Longitudinal Study on Aging
OR: Odds ratio
RN: Registered nurse
VIF: Variance inflation factor
